# Intraluminal micro-stenting improves urinary tract reconstruction in murine kidney transplant model

**DOI:** 10.1186/s42826-026-00301-w

**Published:** 2026-10-14

**Authors:** Zi-Jie Xu, Long He, Zheng Tang, Ren-Jie Wang, Yin Yang, Kai-Ying Chen, Ze-Tao Ding, Jun-Ru Lu, Hao-Dong Chi, Ji-Fu Ge, Zhi-Hong Liu

**Affiliations:** 1 Department of Urology, Shanghai General Hospital, Shanghai Jiao Tong University School of Medicine, No.100, Haining Road, Hongkou District, Shanghai, China; 2 Organ Transplant Center, General Hospital of Northern Theater Command, Shenyang, China

**Keywords:** Kidney transplantation, Animal models, Ureteral reconstruction, Ureteral stenting, Postoperative complications

## Abstract

**Background:**

Urinary tract reconstruction is a key technical barrier in murine kidney transplantation due to the tiny ureteral caliber. Conventional methods are technically demanding with high morbidity. This study assesses a refined intraluminal stenting approach for better surgical stability, fewer complications, and preserved anti-reflux structural anatomy of the ureterovesical junction. We performed 88 orthotopic mouse kidney transplantations using three distinct reconstruction modalities: bladder patch (*n* = 31), ureter implantation (*n* = 28), and intraluminal stenting (*n* = 29). The stenting protocol utilized a micro-polyethylene (PE) stent to achieve end-to-end ureteral alignment. Outcomes included operative efficiency, learning curve trajectories, postoperative survival, and urinary-related morbidity.

**Results:**

The stenting cohort achieved a 14-day survival rate of 96.6% (28/29), significantly higher than the bladder patch group (67.7%, 21/31; *p* = 0.006), while the difference compared with the ureter implantation group (82.1%, 23/28) did not reach statistical significance (*p* = 0.102). Intraluminal stenting markedly reduced reconstruction time (8‘22’‘±0’41’’, *p* < 0.05 vs. both control groups) and was associated with an accelerated proficiency acquisition curve. The overall urological morbidity in the stenting group was 10.3% (3/29), consisting of 1 case of mild urinary leakage and 2 cases of mild hydronephrosis, which was significantly lower than in the bladder patch (58.1%, 18/31) and ureter implantation (42.9%, 12/28) cohorts (*p* < 0.05 for morbidity comparison). Notably, this approach effectively precluded severe complications including ischemic necrosis, ureteral dislodgement, and obstruction, with no urinary tract reconstruction-related deaths observed.

**Conclusions:**

This retrospective study shows that the intraluminal stenting technique offers a reliable and highly reproducible approach for urinary tract reconstruction in murine kidney transplantation. This modality significantly minimizes technical complexity and postoperative morbidity, and maintains the intact ureterovesical anatomy that provides the structural foundation for physiological anti-reflux function. It provides an optimized surgical platform for short-term experimental protocols, particularly in acute rejection research.

**Graphical Abstract:**

**Figure:**
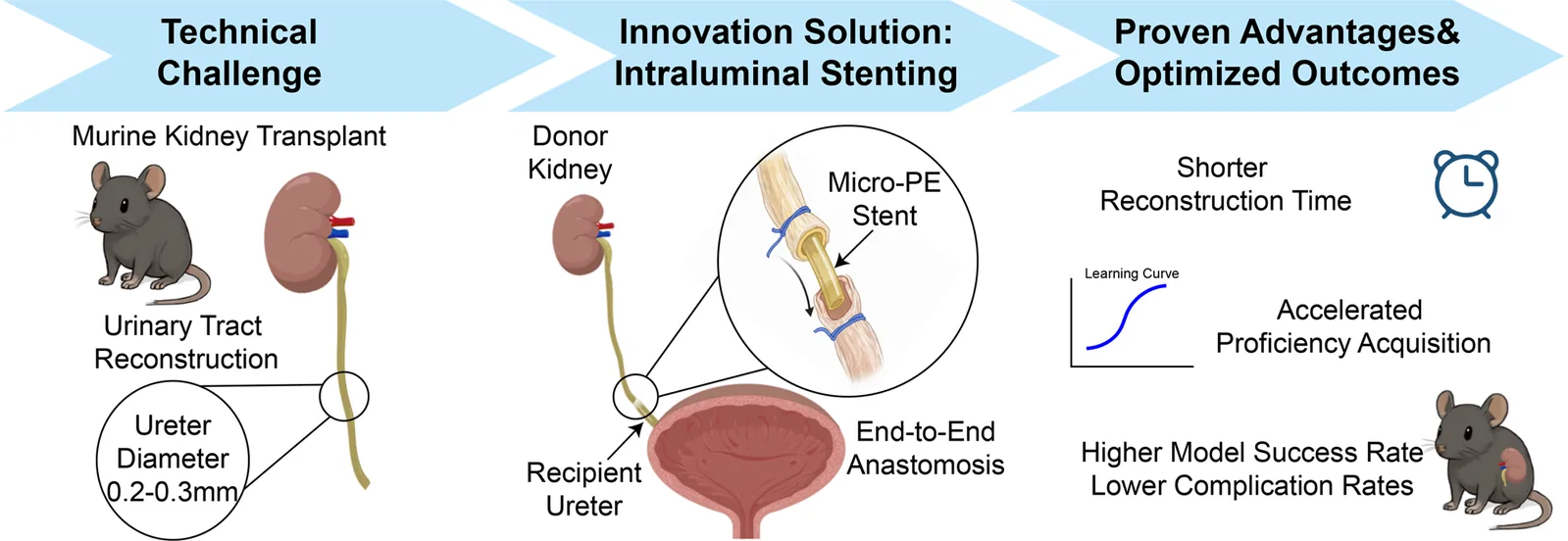

**Supplementary Information:**

The online version contains supplementary material available at https://doi.org/10.1186/s42826-026-00301-w.

## Background

The murine kidney transplant model is a fundamental tool for investigating ischemia-reperfusion injury (IRI) and transplant immunomodulation [1, 2]. It is also essential for studying mechanisms of acute and chronic rejection [3]. However, the procedure is technically demanding. It requires microsurgical anastomosis of vessels approximately 1 mm in diameter. Furthermore, complex urinary tract reconstruction is necessary. Conventional suture-only end-to-end anastomosis is often unfeasible in mice due to the minute ureteral diameter. Consequently, reconstruction represents a primary technical barrier. This step ultimately determines the success rate, stability, and reproducibility of the model [4].

Currently, the two dominant techniques are the bladder patch method and ureter implantation [5, 6]. The bladder patch technique was the first standardized approach for urinary tract reconstruction. This method provides anatomical anti-reflux advantages for long-term observation. However, it remains technically demanding and associated with significant postoperative morbidity. The bladder patch is particularly susceptible to ischemic necrosis [7, 8]. When using BALB/c donors, the incidence of cuff necrosis and urinary leakage can reach 50% [9]. In contrast, the ureter implantation technique streamlines the procedure. This efficiency allows reconstruction to be completed within 10 minutes [10, 11]. Consequently, this method has gained broad adoption. However, it is highly operator-dependent. The puncture site size, implantation length, and fixation method significantly influence outcomes [12]. For instance, an inappropriate puncture size can lead to ureteral stenosis or anastomotic leakage. Excessive residual ureteral or mesenteric tissue within the bladder may also lead to necrosis and infection. Sloughed necrotic tissue often causes late-stage urinary obstruction. Furthermore, inadequate fixation or distal ischemia frequently results in urinary leakage. The micro-bladder patch technique, developed as a hybrid approach, unfortunately inherits the limitations of its predecessors. It carries the risk of cuff necrosis without resolving the issues of obstruction and infection [13]. Alternatively, intraureteral catheterization—where the recipient’s ureter is inserted into the donor renal pelvis—preserves anti-reflux function and reduces operative time. However, this method requires an invasive incision into the donor pelvis [14]. This causes iatrogenic damage to the renal parenchyma. It also relies on the recipient’s ischemic, disused ureter, which increases the long-term risk of stenosis.

Notably, most existing techniques disrupt the native ureterovesical junction (UVJ) structure. The ureter implantation method bypasses the physiological anti-reflux mechanism of the bladder wall, predisposing recipients to vesicoureteral reflux, recurrent upper urinary tract infection, and progressive hydronephrosis. Although the bladder patch method preserves certain anti-reflux properties, it comes at the cost of high ischemic necrosis risk. Consequently, all existing techniques for urinary tract reconstruction remain suboptimal. Significant technical complexity, inconsistent success rates, and compromised urinary tract physiology preclude the widespread application of this model. Therefore, the current landscape necessitates a reconstruction protocol that is both efficient and highly reproducible, while preserving the native anti-reflux anatomy. Such an advancement would overcome existing technical barriers and provide a more reliable experimental platform for transplant immunology.

In this study, we introduce a refined intraluminal stenting technique for end-to-end ureteral anastomosis. This approach utilizes a micro-stent to achieve rapid and reliable urinary tract reconstruction, while fully retaining the recipient’s intact ureterovesical junction. We systematically evaluated the surgical workflow, learning curve, and postoperative outcomes of this method. By comparing this technique with conventional modalities, we aim to provide an optimized technical solution. This advancement facilitates the broader adoption and standardization of the murine kidney transplant model.

## Methods

### Animals

Specific pathogen-free (SPF)-grade male C57BL/6 and BALB/c mice (weight 22–25 g) were obtained from Shanghai Model Organisms Center, Inc. For all three experimental groups, BALB/c mice served as organ donors and C57BL/6 mice served as recipients, establishing a fully major histocompatibility complex (MHC)-mismatched acute rejection model. Strain allocation was uniform across all reconstruction groups, with no intergroup difference in donor/recipient strain combination; grouping was based solely on the urinary tract reconstruction technique. Animals were housed in an SPF-grade facility under controlled conditions with ad libitum access to food and water. All procedures were performed in accordance with the *Guide for the Care and Use of Laboratory Animals* and approved by the Animal Ethics Committee of Shanghai General Hospital (Approval No. 2025AWS353).

### Study design

This retrospective study consecutively collected orthotopic mouse kidney transplant cases performed at our center between March 2024 and December 2025. A total of 94 consecutive procedures were performed during the study period. The primary analysis included 88 cases that met the predefined criterion of survival > 3 days after bilateral native nephrectomy. Cases with intraoperative death, vascular anastomosis failure, or all-cause death within 3 days postoperatively were excluded to avoid confounding from non-urinary reconstruction-related perioperative events on the endpoint evaluation. These eligible cases were divided into three groups according to the urinary reconstruction technique: the Intraluminal Stenting group (*n* = 29), the Bladder patch group (*n* = 31), and the Ureter implantation group (*n* = 28). All surgical procedures were performed by a single senior microsurgeon, thereby completely eliminating inter-surgeon heterogeneity in technical proficiency. Prior to the study, the surgeon had completed more than 50 mouse heart transplant procedures with stable microsurgical skills but had no prior experience in mouse urinary tract reconstruction. To minimize confounding from the surgeon’s cumulative proficiency gain, the three techniques were applied in a fixed sequential cyclic order (Stenting → Bladder patch → Ureter implantation) across all consecutive cases. Throughout the study, all key parameters of the three techniques, as well as non-technical aspects including the anesthesia regimen, surgical instruments, vascular anastomosis protocol, and postoperative care, were fully standardized.

### Reagents and equipment

Surgical procedures were performed using a Zeiss stereomicroscope (Carl Zeiss, Germany). Microsurgical instruments, including curved micro-forceps (504155), microvascular clamps (501779 G), Adson forceps (14226), and Bonn forceps (504475), were sourced from World Precision Instruments (WPI, USA). Nylon sutures (9–0 and 10–0; VT04A00N07 13) were obtained from Arosurgical Instruments Corp (USA). The 28.5G needles were purchased from WPI. The intraluminal stents were fabricated from medical-grade polyethylene (PE). These stents featured an outer diameter of 0.1 mm, an inner diameter of 0.05 mm, and a total length of 2 mm. Both ends were polished into smooth bevels and sterilized with ethylene oxide prior to use. General anesthesia was induced via intraperitoneal injection of a ketamine (80 mg/kg) and xylazine (5 mg/kg) mixture, consistent with the donor surgery protocol. Additional reagents, including povidone-iodine, sterile saline, and heparin sodium, were utilized throughout the procedures.

### Donor kidney harvest

BALB/c donor mice were anesthetized via intraperitoneal (i.p.) injection of ketamine (80 mg/kg) and xylazine (5 mg/kg) diluted in normal saline. After standard skin preparation and disinfection, a midline abdominal incision was made to expose the left kidney and hilar vessels. The entire length of the ureter was carefully mobilized, ensuring the preservation of the peri-ureteral mesentery and blood supply, and then transected distally. The left renal vein, abdominal aorta, and inferior vena cava (IVC) branches were isolated. Small branches, including the adrenal and gonadal vessels, were ligated and divided to prevent hemorrhage. The kidney was perfused in situ through the infrarenal aorta with ice-cold heparinized saline (100 U/ml) until the organ blanched uniformly. The left kidney was then harvested with the abdominal aorta and renal vein attached and stored in 4 °C saline.

### Establishment of the kidney transplant Model

#### Intraluminal stenting for urinary reconstruction

**Fig. 1:**
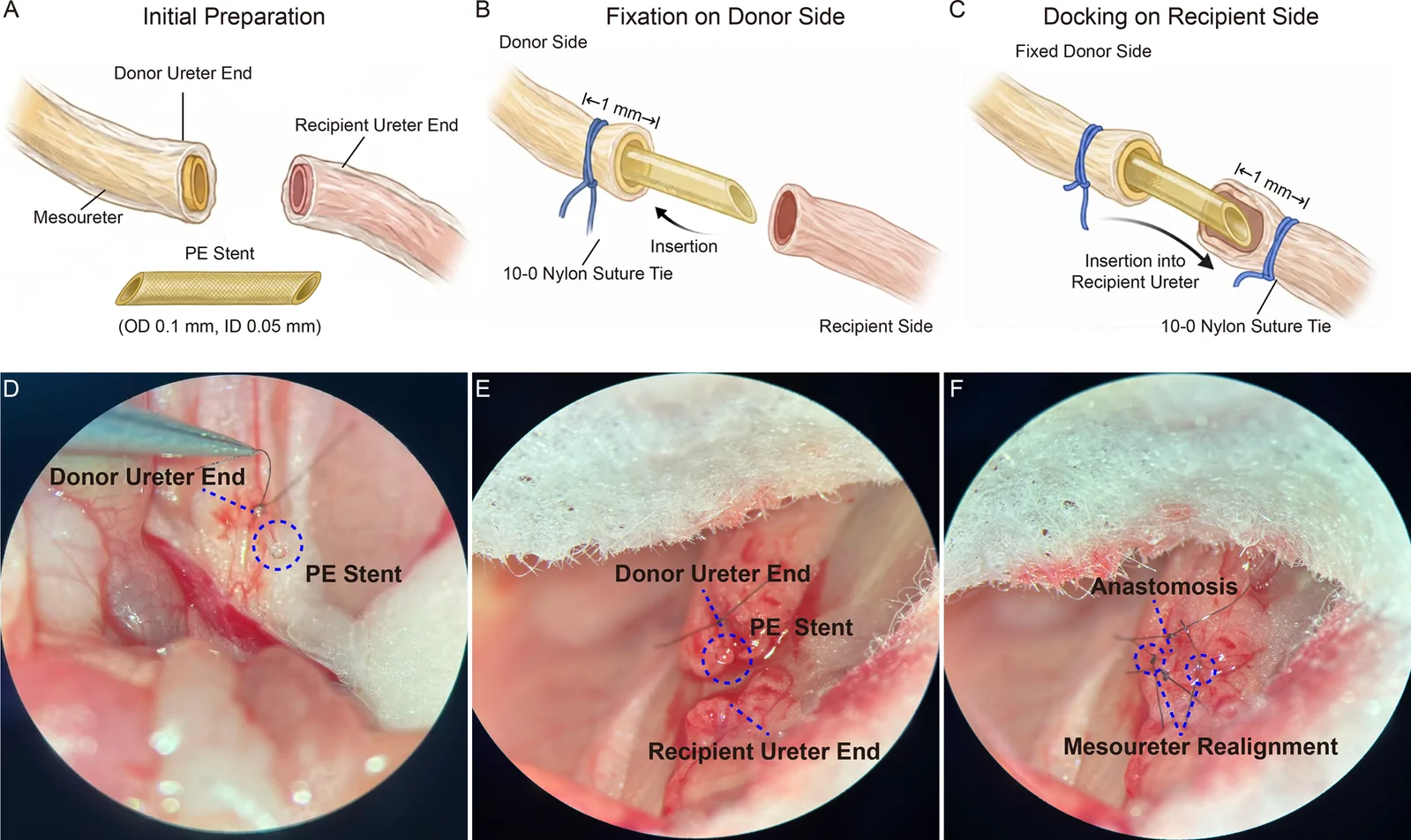
Surgical procedure of the novel intraluminal stenting technique for ureteral end-to-end anastomosis in mice. (**A**) Schematic representation: the donor and recipient ureteral ends are aligned with the medical-grade polyethylene (PE) stent (outer diameter (OD): 0.1 mm, inner diameter (ID): 0.05 mm, length: 2 mm). (**B**) Donor-side fixation: the PE stent is inserted 1 mm into the donor ureter and secured with a 10–0 nylon circumferential tie. (**C**) Recipient-side docking: the remaining end of the stent is inserted into the recipient ureter and secured with a second 10–0 nylon tie, completing the end-to-end anastomosis. (D–F) intraoperative microphotographs: (**D**) Insertion of the beveled PE stent into the donor ureteral lumen. (**E**) Alignment and docking of the stent into the recipient ureter. (**F**) The finalized reconstruction showing restored anatomical continuity and precise realignment of the ureteral mesentery

Following anesthesia and preparation, a midline incision was made in the recipient. Bilateral native nephrectomy was performed, with the left ureter completely resected. An 8 mm segment of the right ureter was retained, with the proximal end transected at the native renal pelvis and the distal end preserved at the bladder junction, for end-to-end anastomosis with the donor ureter. The recipient’s abdominal aorta and IVC were mobilized, and microvascular clamps were placed proximally and distally to the planned anastomosis site. End-to-side anastomoses were performed between the donor’s aortic cuff and the recipient’s aorta, and between the donor’s renal vein and the recipient’s IVC using 10–0 nylon sutures. After removing the clamps, the kidney was inspected for adequate perfusion and hemostasis. Ureteral reconstruction via the intraluminal stenting technique was then performed under the microscope (Fig. 1A). One end of the sterile PE stent (with 45° beveled ends) was gently inserted approximately 1 mm into the distal end of the donor ureter. The stent was secured with a 10–0 nylon circumferential tie to prevent dislodgement or ureteral twisting (Fig. 1B, D). This step can also be performed during donor harvest to facilitate insertion. The remaining end of the stent was then inserted approximately 1 mm into the proximal end of the recipient ureter. After ensuring the ureter was properly aligned without tension or torsion, it was secured with another 10–0 nylon tie to complete the end-to-end connection (Fig. 1C, E). Finally, the corresponding donor and recipient ureteral mesenteries were aligned and secured with a single interrupted 10–0 suture on each side (Fig. 1F). This step restores the anatomical position, protects the blood supply, and reduces tension at the anastomosis. After confirming patency and the absence of leakage via gentle pressure on the graft, the abdomen was closed in layers.

#### Bladder patch technique

The recipient bladder dome was fully exposed. A small incision was created using micro-scissors to match the size of the donor bladder patch. The bladder lumen was then irrigated with sterile saline. Using 10–0 non-traumatic nylon sutures, the donor patch was approximated to the recipient bladder opening via full-thickness interrupted sutures. Specific care was taken to preserve the cuff blood supply and minimize tension. After confirming a watertight anastomosis, the abdominal cavity was rinsed and closed in layers.

#### Ureter implantation technique

The anterior bladder wall of the recipient was exposed. Under the microscope, micro-forceps were used to perform a blunt puncture through the anterior and posterior walls. Sharp injury was strictly avoided. The micro-forceps were passed through the anterior site and out the posterior site to grasp the distal donor ureter. The ureter was then retracted into the bladder lumen and through the anterior puncture site. The pathway was inspected to ensure it was free of tension or torsion. The root of the ureteral mesentery was anchored to the posterior puncture site with a 10–0 nylon suture to prevent dislodgement. Excess ureter at the anterior wall was trimmed. The remaining distal end was gently repositioned into the bladder lumen. Finally, the anterior puncture site was closed with a 10–0 nylon suture. After confirming the absence of leakage, the abdomen was rinsed and closed.

### Postoperative evaluation

All mice were housed individually in a temperature-controlled environment with ad libitum access to food and water. Observations were recorded as follows:

#### Learning curve assessment and statistical analysis

The urinary reconstruction time was defined as the interval from the completion of vascular anastomosis (restoration of graft blood flow) to the completion of urinary tract reconstruction (confirmed patency and absence of leakage). The cumulative sum (CUSUM) method was applied to quantify the learning curve of each technique [15].

#### Survival and mortality analysis

Recipient survival was monitored for 14 days postoperatively, and Kaplan-Meier curves were generated. For mice that died during the observation period, immediate autopsies were performed to determine the cause of death. Deaths were categorized as urinary-related (e.g., bladder necrosis, severe leakage, or obstructive renal failure) or non-urinary-related (e.g., vascular thrombosis/hemorrhage, abdominal infection, or anesthetic accidents).

#### Assessment of urinary complications

Mice were monitored daily for mental status, activity, abdominal signs, and voiding. On postoperative day 14, a systematic autopsy was performed to evaluate complications, including urinary leakage, bladder necrosis, hydronephrosis, ureteral stenosis/obstruction, and bladder stones. Mice exhibiting abnormal signs, such as abdominal distension or lethargy, underwent immediate exploratory dissection.

### Statistical analysis

Data were analyzed with Statistical Package for the Social Sciences (SPSS) 26.0, and visualized via R 4.2.0 (ggplot2). Normally distributed quantitative data were presented as mean ± standard deviation (SD), with overall intergroup comparison by one-way analysis of variance (ANOVA), followed by pre-specified pairwise comparisons using the least significant difference (LSD) test. Non-normally distributed quantitative data were analyzed by Kruskal-Wallis H test for overall difference, with pairwise comparisons using Dunn’s test. Categorical data were presented as counts (percentages) and compared by Pearson χ^2^ test or Fisher’s exact test as appropriate. Pre-specified pairwise comparisons were limited to the intraluminal stenting group versus each of the two control groups, with no comparison between the two control groups. The cumulative sum (CUSUM) method was applied to quantify the learning curve of each technique and objectively identify the inflection point between the learning phase and proficiency plateau. Survival was analyzed by the Kaplan-Meier method with log-rank test.

## Results

### Comparison of operative durations and learning curves

**Fig. 2:**
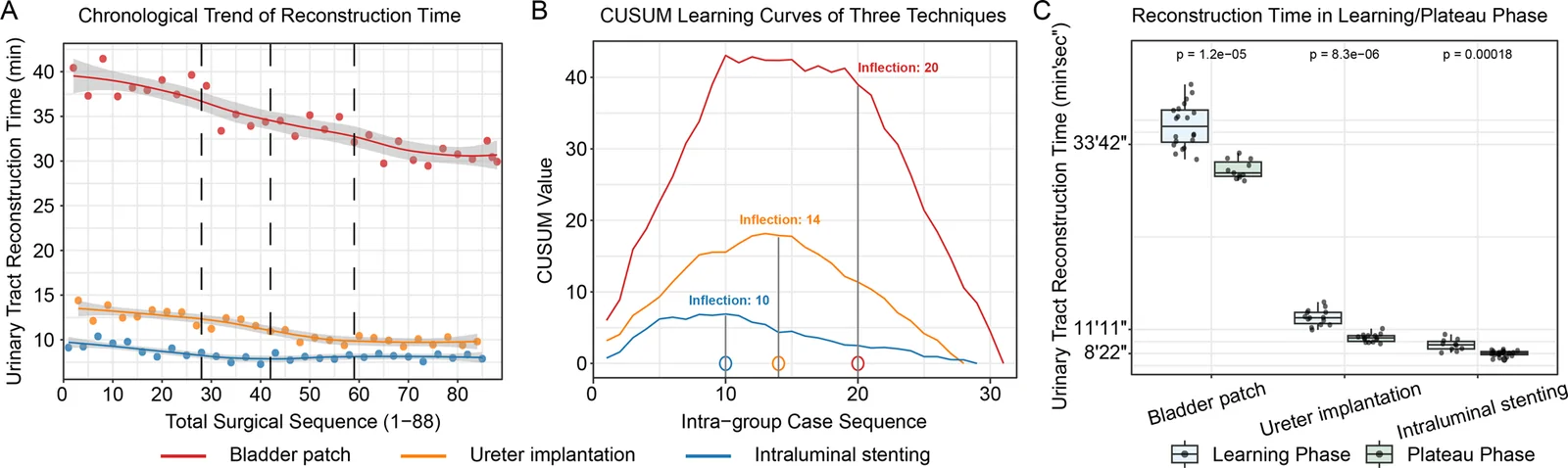
Learning curve of urinary tract reconstruction time. (**A**) Chronological trend of urinary tract reconstruction time for the three techniques along the overall surgical sequence. (**B**) CUSUM learning curves of the three urinary tract reconstruction techniques, with the peak of each curve marking the objective inflection point between the learning phase and the proficiency plateau phase. (**C**) Box plot of urinary tract reconstruction time in the learning phase and plateau phase of the three techniques

Baseline characteristics of donor and recipient mice were well balanced across groups (Table S1*Baseline characteristics of donor and recipient mice in the three groups*, *p* > 0.05). A total of 88 successful mouse orthotopic kidney transplant models were analyzed, including 29 in the intraluminal stenting group, 31 in the bladder patch group, and 28 in the ureter implantation group (Table S2*Chronological recording of urinary tract reconstruction times for the three experimental groups*). The intraluminal stenting technique achieved the shortest urinary tract reconstruction time (overall mean: 8′22′′±0′41′′), significantly lower than the bladder patch group (33′42′′±3′37′′) and ureter implantation group (11′11′′±1′25′′, *p* < 0.05, Table 1). All three techniques exhibited a progressive reduction in reconstruction time with increasing operative volume, but differed markedly in learning efficiency (Fig. 2A, B). The intraluminal stenting group reached proficiency after 10 cases, with mean time decreasing from 9′05′′±0′41′′ (learning phase) to 7′59′′±0′29′′ (plateau phase; 12.3% reduction). The ureter implantation group required 14 cases to plateau, with mean time falling from 12′09′′±1′05′′ to 10′14′′±0′32′′ (15.9% reduction). The bladder patch group had the longest learning curve, plateauing after 20 cases, with mean time declining from 36′40′′±2′54′′ to 28′19′′±1′10′′ (22.8% reduction). Box plot analysis further validated the stability of the plateau phase across all groups (Fig. 2C). These findings demonstrate that the intraluminal stenting technique not only minimizes operative duration but also offers a significantly shorter learning curve and faster technical proficiency compared with the bladder patch and ureter implantation techniques.

**Table 1:** Comparison of surgical duration and learning curve characteristics among the three urinary tract reconstruction techniques

Group	Phase Classification (Based on Objective CUSUM Inflection Point)	Number of Cases (n)	Urinary Tract Reconstruction Time (xˉ±s, min’sec’’)	Intra-group Reduction in Duration	Inter-group Overall Comparison p-value
Intraluminal stenting	Learning phase (Cases 1–10)	10	9‘05’‘± 0’41’’	12.30%	<0.001^*#^
	Plateau phase (Cases 11–29)	19	7‘59’‘± 0’29’’	-	-
	Overall	29	8‘22’‘± 0’41’’	-	-
Bladder patch	Learning phase (Cases 1–20)	20	36‘40’‘± 2’54’’	22.80%	Reference group
	Plateau phase (Cases 21–31)	11	28‘19’‘± 1’10’’	-	-
	Overall	31	33‘42’‘± 3’37’’	-	-
Ureter implantation	Learning phase (Cases 1–14)	14	12‘09’‘± 1’05’’	15.90%	<0.001^*^
	Plateau phase (Cases 15–28)	14	10‘14’‘± 0’32’’	-	-
	Overall	28	11‘11’‘± 1’25’’	-	-

Notes: *p < 0.05 vs. Bladder patch group; #p < 0.05 vs. Ureter implantation group (Kruskal-Wallis H test with Dunn-Bonferroni correction for multiple comparisons)

Intra-group reduction in duration = [(Mean learning phase duration − Mean plateau phase duration)/Mean learning phase duration] × 100%

### Postoperative survival and mortality analysis

**Fig. 3:**
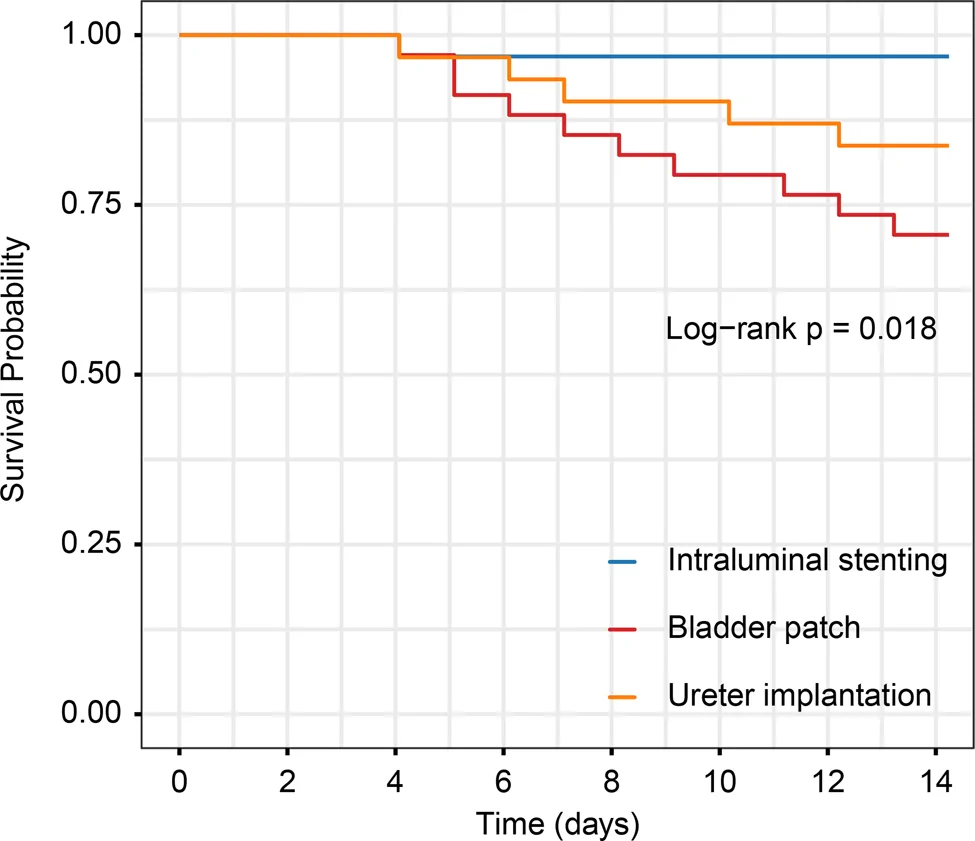
Postoperative 14-day survival curve of mice in three groups

We generated 14-day Kaplan-Meier survival curves for the three ureteral reconstruction techniques (Fig. 3). Log-rank test showed statistically significant difference in survival among the three cohorts (*p* = 0.018). The intraluminal stenting group achieved a 96.6% 14-day overall survival rate (28/29). Only one perioperative death was recorded in this group, which was attributed to delayed bleeding at the vascular anastomosis. Critically, no deaths related to urinary tract reconstruction were observed in this cohort. In comparison, the bladder patch group had a significantly lower 14-day overall survival rate of 67.7% (21/31, *p* = 0.006 vs. the intraluminal stenting group, Fisher’s exact test). A total of 10 fatal events occurred in this cohort. One death was due to delayed vascular anastomotic bleeding, a common surgical event observed across all three groups, unrelated to ureteral reconstruction. The remaining 9 deaths were directly caused by severe complications secondary to urinary tract reconstruction. These included lethal peritonitis from anastomotic urinary leakage (*n* = 3), ischemic necrosis of the bladder patch (*n* = 4), and obstructive renal failure caused by chronic urinary retention (*n* = 2). The ureter implantation group showed an intermediate 14-day survival rate of 82.1% (23/28). The difference compared with the intraluminal stenting group did not reach statistical significance (*p* = 0.102, Fisher’s exact test). A total of 5 fatal events occurred in this cohort. One death was attributed to delayed vascular anastomotic bleeding, consistent with the other groups. The remaining 4 deaths were all caused by adverse events related to urinary reconstruction. The specific etiologies included fatal peritonitis from urinary leakage (*n* = 3), and obstructive renal failure due to ureteral stump necrosis (*n* = 1) (Table 2).

**Table 2:** Survival, mortality and death time among three groups

Group	Total cases(n)	Deaths (n)	Survival (n)	Survival rate (%)	Time to death (days)
Intraluminal stenting	29	1	28	96.6	4
Bladder patch	31	10	21	67.7^*^	4, 5, 5, 6, 7, 8, 9, 11, 12, 13
Ureter implantation	28	5	23	82.1	4, 6, 7, 10, 12
Total	88	16	72	81.8	-

Note: *p = 0.006 vs. the intraluminal stenting group (two-sided Fisher’s exact test). The survival difference between the ureter implantation group and the intraluminal stenting group was not statistically significant (p = 0.102)

## Evaluation of urinary-related complications

**Fig. 4:**
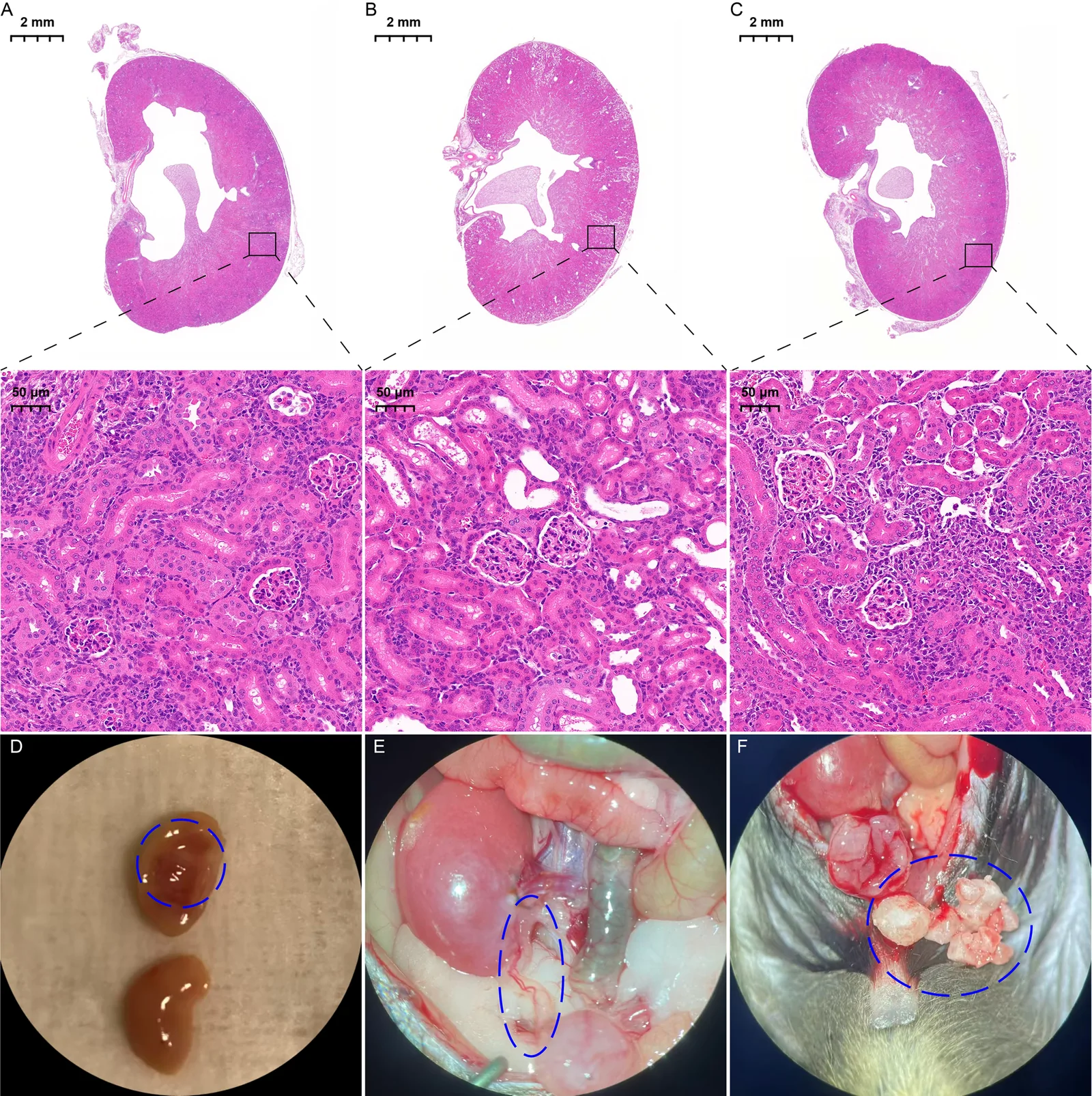
Pathological manifestations and surgical complications following urinary tract reconstruction in mouse renal transplantation. (A–C) HE staining images of renal pelvic morphology from the ureter implantation, bladder patch, and intraluminal stenting groups in sequence. (**D**) Severe hydronephrosis in the ureter implantation group. (**E**) Ureteral obstruction in the ureter implantation group. (**F**) Multiple large bladder stones identified at 1 month post-surgery in an animal under extended observation

**Fig. 5:**
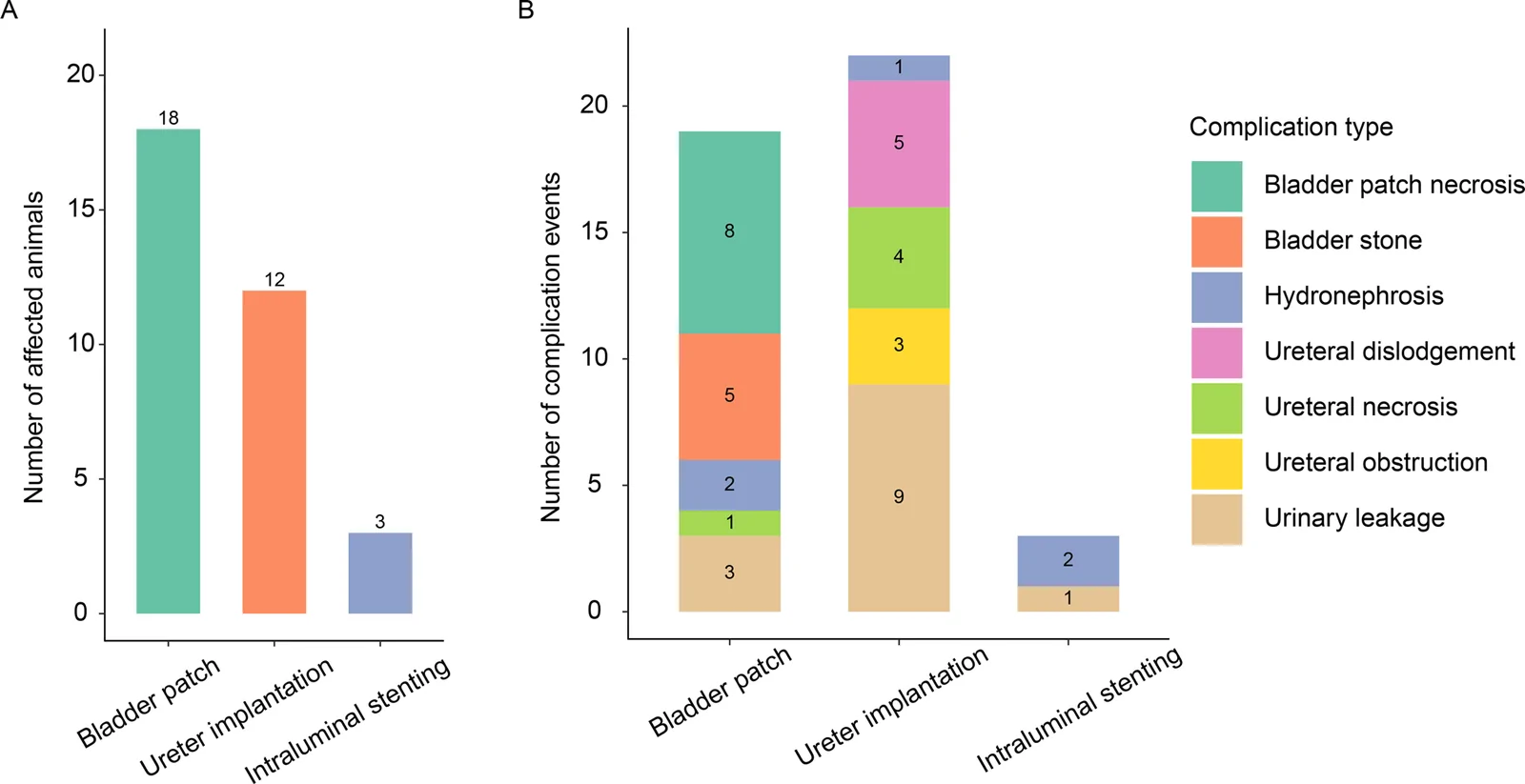
Comparison of postoperative urinary complications across the three surgical groups. (**A**) Number of affected animals (subjects with at least one complication) per group. (**B**) Total number of complication events stratified by subtype. Note: animals with multiple concurrent complications are counted once in Panel **A** but multiple times in Panel **B**

We defined the overall complication rate as the proportion of subjects with at least one urinary adverse event. Subjects with multiple concurrent complications were counted only once to avoid overestimation (Table 3). Urinary leakage was the most common acute perioperative complication. The ureter implantation group had the highest leakage incidence (9/28, 32.1%), followed by the bladder patch group (3/31, 9.7%). The intraluminal stenting group had only 1 case of mild, self-limiting urinary leakage (1/29, 3.4%), which resolved with conservative management. Postmortem pathological examination further verified obvious intergroup discrepancies. Hematoxylin and eosin (HE) staining demonstrated severe rejection reactions with no significant intergroup differences (Fig. 4A–C). Marked pelvic dilatation was observed in the ureter implantation group (Fig. 4A), mild pelvic dilatation in the bladder patch group (Fig. 4B), whereas the intraluminal stenting group exhibited renal pelvic morphology closest to normal (Fig. 4C), indicating better preservation of normal renal pelvic morphology. In the bladder patch group, 19 complication events occurred in 18 affected mice, including 1 case with overlapping necrosis and leakage (consistent with Table 3). Complications included bladder patch necrosis (*n* = 8), ureteral necrosis (*n* = 1), bladder stones (*n* = 5), and hydronephrosis (*n* = 2); no ureteral obstruction or ureteral dislodgement was observed in this group. Of the 8 mice with bladder patch necrosis, 4 cases progressed to severe ischemic injury and fatal postrenal renal failure, while the remaining 4 cases with mild focal necrosis survived to the 14-day observation endpoint without fatal sequelae. In the ureter implantation group, 22 complication events occurred in 12 affected mice. Ten mice had multiple complications, including 9 cases with necrosis or dislodgement combined with leakage, and 1 case with obstruction and hydronephrosis (Fig. 4D), which was consistent with Table 3. Complications included ureteral necrosis (*n* = 4), ureteral dislodgement (*n* = 5), ureteral obstruction (*n* = 3, Fig. 4E), and hydronephrosis (*n* = 1); no bladder patch necrosis or bladder stones were detected in this group. Of the 4 mice with ureteral necrosis and 3 mice with ureteral obstruction, only 1 case progressed to complete obstructive renal failure and death, while the other 6 cases maintained partial ureteral patency and survived the observation period. In contrast, the intraluminal stenting group exhibited a low complication profile. Only 3 adverse events were observed in 3 mice: 1 case of urinary leakage and 2 cases of hydronephrosis. No tissue necrosis, ureteral dislodgement, ureteral obstruction, bladder patch necrosis, or bladder stones were detected. The total complication rate was 10.3% (3/29) in the intraluminal stenting group, 58.1% (18/31) in the bladder patch group, and 42.9% (12/28) in the ureter implantation group. The rate in the stenting group was significantly lower than in the other two groups (*p* < 0.05 vs. the bladder patch and ureter implantation groups, Fisher’s exact test with Bonferroni correction, Table 3). Figure 5 presents the complication findings in two complementary panels: Panel A shows the number of animals with at least one urinary complication in each group, and Panel B displays the total number of discrete complication events stratified by subtype across the three cohorts. However, in one mouse from the stenting group that underwent extended observation beyond the 14-day study endpoint, extensive idiopathic bladder calculi were found at 1 month post-surgery (Fig. 4F), the underlying cause of which remains undetermined. This intraluminal stenting technique is primarily recommended for short-term experimental observations, such as research on acute rejection within the initial two weeks post-transplantation.

**Table 3:** Comparison of postoperative complication rates among three groups

Group	Cases	Urinary leakage	Ureteral obstruction	Hydronephrosis	Ureteral necrosis	Bladder patch necrosis	Bladder stone	Ureteral dislodgement	No. of affected animals	Total incidence
Intraluminal stenting	29	1	0	2	0	0	0	0	3	10.3%^*^
Bladder patch	31	3	0	2	1	8	5	0	18	58.1%
Ureter implantation	28	9	3	1	4	0	0	5	12	42.9%

Note: Data for “No. of affected animals” represent the number of affected subjects. In the bladder patch group, 19 complication events occurred across 18 mice (one case of overlapping necrosis and leakage). In the ureter implantation group, 22 events occurred across 12 mice, as 10 subjects presented with multiple concurrent complications (9 cases of necrosis or dislodgement combined with leakage; 1 case of obstruction with hydronephrosis). ^*^ *p* < 0.05 vs. the bladder patch and ureter implantation groups (Fisher’s exact test with Bonferroni correction)

## Discussion

Mouse kidney transplantation is a fundamental tool for studying transplant immunology and ischemia-reperfusion injury [16, 17]. However, urinary tract reconstruction remains a primary technical barrier. The minute diameter of the mouse ureter (only 0.2–0.3 mm) makes conventional suture-based end-to-end anastomosis extremely challenging and operator-dependent. Established modalities are often characterized by high technical demands, frequent complications, and poor model stability. Collectively, these factors hinder experimental reproducibility [18]. Currently, the most common clinical adaptations—the bladder patch and ureter implantation techniques—possess significant drawbacks. These limitations restrict their utility across diverse research applications. In this study, we introduced a refined intraluminal stenting technique for ureteral end-to-end anastomosis. By combining micro-PE stent support with mesenteric realignment sutures, this method achieves rapid and consistent reconstruction while preserving the recipient’s intact ureterovesical anti-reflux anatomy. Our results demonstrate clear practical advantages in operative efficiency and postoperative outcomes.

Operative efficiency and the associated learning curve are critical metrics for evaluating the utility and scalability of any animal model [19]. Our retrospective analysis of 88 successful cases revealed that the mean reconstruction time in the intraluminal stenting group was 8'22''±0'41''. This was significantly shorter than the durations required for the bladder patch group (33'42''±3'37'') and the ureter implantation group (11'11''±1'25'') (*p* < 0.05), confirming the efficiency of the new approach. Learning curve analysis further indicated that proficiency in the stenting technique was achieved after only 10 successful procedures, at which point the operative time plateaued at 7'59''±0'29''. In contrast, the bladder patch and ureter implantation groups required 20 and 14 successful cases, respectively, to reach stability. This disparity stems from the core technical design of the intraluminal stenting method: it bypasses complex steps such as bladder patch tailoring, bladder puncture, or ureteral pull-through. By utilizing medical-grade PE micro-stents for precise end-to-end alignment, the procedure is significantly simplified. This reduces the dependency on advanced microsurgical dexterity, making the model more accessible to researchers and particularly beneficial for those in the early stages of mastering transplantation techniques.

Postoperative survival and complication rates are decisive factors for the stability and experimental utility of animal models [20], forming the central focus of our comparative evaluation. Our results showed a 14-day survival rate of 96.6% in the intraluminal stenting group (28/29). The only perioperative death in this group was attributed to delayed bleeding at the vascular anastomosis, a common surgical complication unrelated to urinary tract reconstruction. Importantly, no urinary reconstruction related deaths occurred in the stenting group. This survival rate was significantly higher than that of the bladder patch group (67.7%; *p* = 0.006), while the difference compared with the ureter implantation group (82.1%) did not reach statistical significance (*p* = 0.102).

Regarding complications, the intraluminal stenting group recorded an overall incidence of only 10.3% (3/29), consisting of a single case of minor leakage and two cases of mild hydronephrosis. Crucially, no severe or irreversible complications, such as ureteral necrosis, permanent obstruction, or bladder patch necrosis, were observed in this group. This rate was significantly lower than the complication rates in the bladder patch (58.1%) and ureter implantation (42.9%) groups (*p* < 0.05). This performance is intrinsically linked to the technical design of the stenting procedure. The micro-PE stent provides stable intraluminal support, effectively mitigating the risks of anastomotic tension or displacement and thereby reducing leakage. Furthermore, the realignment of the ureteral mesentery preserves the blood supply to the anastomotic segment, significantly lowering the incidence of ischemic necrosis. Together, these features address the fundamental flaws inherent in traditional techniques.

Most notably, the intraluminal stenting technique preserves the complete anatomical structure of the recipient’s ureterovesical junction, which retains the structural basis of physiological anti-reflux function. This is a core advantage over the ureter implantation technique, which disrupts the bladder wall’s anti-reflux barrier and increases the risk of vesicoureteral reflux, ascending infection, and chronic hydronephrosis. In this cohort, only 2 cases of mild, transient hydronephrosis were noted in the stenting group, and the renal pelvic morphology on histological sections of the graft kidneys resembled that of normal kidneys, consistent with preserved anatomy of the anti-reflux mechanism. This physiological advantage not only reduces early postoperative morbidity, but also minimizes confounding factors for transplant immunology studies, as reflux-related renal inflammation can interfere with the interpretation of rejection phenotypes.

Consistent with previous reports, the bladder patch technique, despite its anti-reflux advantages for long-term observation, remains limited by its complexity and high rates of cuff necrosis and leakage—complications that can reach 50% specifically when using BALB/c donors [21]. In our study, this group exhibited 8 cases of bladder patch necrosis and 3 cases of urinary leakage. Among the 10 total deaths, 9 were caused by urinary tract-related complications, including lethal peritonitis secondary to leakage (*n* = 3), ischemic necrosis (*n* = 4), and obstructive renal failure (*n* = 2), confirming the inherent risks of this approach. Similarly, while ureter implantation is relatively simple, it is prone to leakage, dislodgement, and stenosis due to variables such as puncture size and fixation methods [22, 23]. We observed a leakage rate of 32.1% in this group, alongside cases of stump necrosis (*n* = 4), obstruction (*n* = 3), and dislodgement (*n* = 5). Of the 5 total deaths, 4 were related to urinary reconstruction, including fatal peritonitis from leakage (*n* = 3) and obstructive renal failure due to stump necrosis (*n* = 1), further demonstrating the instability of this conventional method. By utilizing a non-invasive intraluminal docking design, our stenting method bypasses the ischemic risks of the bladder patch and the high complication rates of implantation, while maintaining native anti-reflux physiology.

This study has several limitations. First, all procedures were performed by a single surgeon, which eliminates inter-operator heterogeneity but may limit the generalizability of the learning curve results. Second, this study only included BALB/c and C57BL/6 mouse strains, and the applicability of this technique in other strains needs further validation in future studies. Third, this study only focused on 14-day short-term outcomes. The temporal progression of stent-related complications shows that the technique maintains excellent safety with no stone formation or delayed stenosis within 14 days. After 4 weeks of indwelling, chronic irritation from the non-degradable PE stent significantly increases the risk of calculi [24], as shown in our extended observation case. Therefore, this technique is currently most suitable for short-term experimental protocols such as acute rejection studies within 14 days. For experiments requiring 3–4 weeks of observation, potential optimization strategies include: (1) using biodegradable polymer stents that degrade spontaneously within 2–3 weeks; (2) performing minimally invasive stent removal via the urethra at postoperative day 10–14 after anastomotic healing; (3) modifying postoperative hydration management to reduce crystal deposition and stone formation risk. These strategies will be systematically validated in future studies. Fourth, physiological anti-reflux function was not directly assessed via functional assays in this study. The inference of an anti-reflux benefit is based on preserved ureterovesical anatomy and histological renal pelvic morphology, and will require formal functional validation in future work. Future research into more biocompatible materials and precisely tailored stent parameters may help mitigate the risk of late-stage urolithiasis [25] and expand the utility of this promising microsurgical method.

To clarify the respective application scenarios of each technique, we summarize their advantages, limitations, and optimal experimental settings as Table 4.

**Table 4:** Comparison of three urinary tract reconstruction techniques in mouse kidney transplantation

Technique	Advantages	Limitations	Optimal Application
Bladder patch	Anti-reflux; Suitable for long-term observation	Steep learning curve; Long operation time; High necrosis/leakage	Chronic rejection; >4 weeks
Ureter implantation	Moderate difficulty; Shorter operation time	No intact anti-reflux structure; High leakage/dislodgement/stenosis; Operator-dependent	Short-term exploratory studies; Experienced surgeons
Intraluminal stenting	Short learning curve; Fast reconstruction; Low complications; Preserves anti-reflux anatomical structure; High reproducibility	PE stent may cause calculi; Not suitable for long-term observation	Acute rejection; IRI; ≤14 days; Ideal for new labs

## Conclusions

In conclusion, this retrospective study shows that the intraluminal stenting technique can effectively optimize urinary tract reconstruction in mouse kidney transplantation. With micro-PE stents and mesenteric realignment, this method significantly shortens operative time, flattens the learning curve, and improves postoperative short-term survival by minimizing urinary-related complications. Most importantly, it preserves the intact ureterovesical anatomy that provides the structural foundation of native anti-reflux function, reducing the risk of hydronephrosis and reflux-related renal injury. This technique is particularly suitable for short-term experimental studies such as acute rejection models within 14 days, and provides a reliable, easy-to-standardize alternative for urinary tract reconstruction in mouse kidney transplantation.

## Electronic supplementary material

Below is the link to the electronic supplementary material.

Supplementary material 1

Supplementary material 2

## Data Availability

The data that support the findings of this study are available from the corresponding author upon reasonable request.

## Abbreviations

AI: Artificial intelligence
ANOVA: Analysis of variance
CUSUM: Cumulative sum
HE: Hematoxylin and eosin
IACUC: Institutional Animal Care and Use Committee
ID: Inner diameter
i.p.: Intraperitoneal
IRI: Ischemia-reperfusion injury
IVC: Inferior vena cava
LSD: Least significant difference
MHC: Major histocompatibility complex
OD: Outer diameter
PE: Polyethylene
SD: Standard deviation
SPF: Specific pathogen-free
SPSS: Statistical Package for the Social Sciences
UVJ: Ureterovesical junction
WPI: World Precision Instruments

## References

[1] Tse GH, Hughes J, Marson LP. Systematic review of mouse kidney transplantation. Transpl Int. 2013;26(12):1149–60. 10.1111/tri.12129 23786597

[2] Barbosa ACS, Mauroner LG, Kumar J, Sims-Lucas S. Delayed graft function post renal transplantation: a review on animal models and therapeutics. Am J Physiol Renal Physiol. 2023;325(6):F817–25. 10.1152/ajprenal.00146.2023 PMC10878700 37855040

[3] Tse GH, Hesketh EE, Clay M, Borthwick G, Hughes J, Marson LP. Mouse kidney transplantation: models of allograft rejection. J Vis Exp. 2014;92:e52163. 10.3791/52163 PMC4540093 25350513

[4] Martins PN. Learning curve, surgical results and operative complications for kidney transplantation in mice. Microsurgery. 2006;26(8):590–93. 10.1002/micr.20292 17066406

[5] Skoskiewicz M, Chase C, Winn HJ, Russell PS. Kidney transplants between mice of graded immunogenetic diversity. Transpl Proc. 1973;5(1):721–25. 4121181

[6] Han WR, Murray-Segal LJ, Mottram PL. Modified technique for kidney transplantation in mice. Microsurgery. 1999;19(6):272–74. 10.1002/(sici)1098-2752(1999)19:6<272::aid-micr3>3.0.co;2-l 10469441

[7] Wang JJ, Hockenheimer S, Bickerstaff AA, Hadley GA. Murine renal transplantation procedure. J Vis Exp. 2009;29:1150. 10.3791/1150 PMC2762912 19593327

[8] Chen H, Zhang Y, Zheng D, Praseedom RK, Dong J. Orthotopic kidney transplantation in mice: technique using cuff for renal vein anastomosis. PLoS One. 2013;8(10):e77278. 10.1371/journal.pone.0077278 PMC3796481 24155935

[9] Zhao DQ, Guo ZL, Pan TH, Chen G. Technical summary and experiences of urinary reconstruction in mouse kidney transplantation. Zhonghua Yi Xue Za Zhi. 2022;102(12):884–88. 10.3760/cma.j.cn112137-20211024-02350 35330583

[10] Plenter R, Jain S, Ruller CM, Nydam TL, Jani AH. Murine kidney transplant technique. J Vis Exp. 2015;105:e52848. 10.3791/52848 PMC4692659 26555373

[11] Plenter RJ, Jain S, Nydam TL, Jani AH. Revised arterial anastomosis for improving murine kidney transplant outcomes. J Invest Surg. 2015;28(4):208–14. 10.3109/08941939.2014.1002641 26087146

[12] Huang LJ, Reese S, Djamali A. Contributing factors to complications and surgical success in mouse kidney transplantation. Int Braz J Urol. 2012;38(3):395–404. 10.1590/s1677-55382012000300013 22765855

[13] Ge X, Timsit MO, Tullius SG. A novel mouse renal transplant technique. Transpl Int. 2011;24(4):e35–7. 10.1111/j.1432-2277.2010.01192.x 21134242

[14] Wang W, Zhang F, Gu B, Hu J, Liu X, Han Z, et al. Application of intraureteral cannula technique in reconstruction of urinary tract in mice kidney transplantation. Zhonghua Shi Yan Wai Ke Za Zhi. 2018;35(9):1706–08.

[15] Woodall WH, Rakovich G, Steiner SH. An overview and critique of the use of cumulative sum methods with surgical learning curve data. Stat Med. 2021;40(6):1400–13. 10.1002/sim.8847 33316849

[16] Snoeijs MG, van Heurn LW, Buurman WA. Biological modulation of renal ischemia-reperfusion injury. Curr Opin Organ Transpl. 2010;15(2):190–99. 10.1097/MOT.0b013e32833593eb 20009928

[17] Zhang ZJ, Casiraghi F, Perkins GB, Baldwin WM 3rd, Fairchild RL. Can mouse kidney transplant models inform mechanisms of injury and acceptance in clinical kidney transplantation? Am J Transpl. 2025;25(7):1391–98. 10.1016/j.ajt.2025.04.001 PMC12221770 40209906

[18] Ge F, Gong W. Strategies for successfully establishing a kidney transplant in a mouse model. Exp Clin Transpl. 2011;9(5):287–94. 21967253

[19] Pusic MV, Boutis K, Pecaric MR, Savenkov O, Beckstead JW, Jaber MY. A primer on the statistical modelling of learning curves in health professions education. Adv Health Sci Educ Theory Pract. 2017;22(3):741–59. 10.1007/s10459-016-9709-2 27699508

[20] Merl S, Chen B, Gunes ME, Atta H, Yang K, Ekanayake-Alper D, et al. Development of a large animal orthotopic intestinal transplantation model with long-term survival for study of immunologic outcomes. Front Transpl. 2024;3:1367486. 10.3389/frtra.2024.1367486 PMC11235311 38993771

[21] Zhang Y, Zheng D, Praseedom R, Zhou H, Huang D, Chen H. Kidney transplantation in mice with intraureteral cannula technique for urinary reconstruction. Transplantation. 2014;98(9):e82–5. 10.1097/TP.0000000000000420 25364869

[22] Rong S, Lewis AG, Kunter U, Haller H, Gueler F. A knotless technique for kidney transplantation in the mouse. J Transpl. 2012;2012:127215. 10.1155/2012/127215 PMC3407654 22852070

[23] Wang ZZ, Wang C, Cunningham EC, Allen RD, Sharland AF, Bishop GA. Optimized method for ureteric reconstruction in a mouse kidney transplant model. ANZ J Surg. 2014;84(6):481–85. 10.1111/ans.12395 24750996

[24] Tomer N, Garden E, Small A, Palese M. Ureteral stent encrustation: epidemiology, pathophysiology. Manag Curr Technol. J Urol. 2021;205(1):68–77. 10.1097/JU.0000000000001343 32856981

[25] Wu Y, Li Z, Wen X, Zhong Y, Chen H, Qian L, et al. Zwitterionic multifunctional coatings with strong hydration for ureteral stents to inhibit infectious encrustation. Bioact Mater. 2025;55:57–73. 10.1016/j.bioactmat.2025.08.015 PMC12481508 41035423

