# Supplementary material for: Intraluminal micro-stenting improves urinary tract reconstruction in murine kidney transplant model
Source: Lab Anim Res. 2026 Oct 14;42:39. doi: 10.1186/s42826-026-00301-w (PMC13663044; doi:10.1186/s42826-026-00301-w)
Supplement: Supplementary file 1 [file 42826_2026_301_MOESM1_ESM.docx]

***Table S1***

***Baseline characteristics of donor and recipient mice in the three groups***

| **Characteristic** | **Intraluminal stenting (n=58)** | **Bladder patch (n=62)** | **Ureter implantation (n=56)** | **P value** |
| --- | --- | --- | --- | --- |
| Body weight (g, x̄±s) | 23.2±0.7 | 23.5±0.8 | 23.3±0.6 | 0.452 |
| Age (weeks, x̄±s) | 8.2±0.4 | 8.3±0.5 | 8.2±0.4 | 0.678 |
| Donor-recipient strain | BALB/c→B6 | BALB/c→B6 | BALB/c→B6 | 1 |

*Note: There were no statistically significant differences in baseline characteristics among the three groups (P > 0.05).*
