# Supplementary material for: Intraluminal micro-stenting improves urinary tract reconstruction in murine kidney transplant model
Source: Lab Anim Res. 2026 Oct 14;42:39. doi: 10.1186/s42826-026-00301-w (PMC13663044; doi:10.1186/s42826-026-00301-w)
Supplement: Supplementary file 2 [file 42826_2026_301_MOESM2_ESM.docx]

***Table S2***

***Chronological recording of urinary tract reconstruction times for the three experimental groups***

| Number | Group | Group number | Time (min) | Time (sec) | Total time (min) |
| --- | --- | --- | --- | --- | --- |
| 1 | Stenting | 1 | 9 | 8 | 9.133 |
| 2 | BladderPatch | 1 | 40 | 26 | 40.433 |
| 3 | Implantation | 1 | 14 | 24 | 14.4 |
| 4 | Stenting | 2 | 9 | 14 | 9.233 |
| 5 | BladderPatch | 2 | 37 | 18 | 37.3 |
| 6 | Implantation | 2 | 12 | 7 | 12.117 |
| 7 | Stenting | 3 | 10 | 23 | 10.383 |
| 8 | BladderPatch | 3 | 41 | 27 | 41.45 |
| 9 | Implantation | 3 | 13 | 52 | 13.867 |
| 10 | Stenting | 4 | 9 | 36 | 9.6 |
| 11 | BladderPatch | 4 | 37 | 14 | 37.233 |
| 12 | Implantation | 4 | 12 | 28 | 12.467 |
| 13 | Stenting | 5 | 9 | 47 | 9.783 |
| 14 | BladderPatch | 5 | 38 | 13 | 38.217 |
| 15 | Implantation | 5 | 12 | 35 | 12.583 |
| 16 | Stenting | 6 | 8 | 38 | 8.633 |
| 17 | BladderPatch | 6 | 37 | 56 | 37.933 |
| 18 | Implantation | 6 | 13 | 19 | 13.317 |
| 19 | Stenting | 7 | 8 | 6 | 8.1 |
| 20 | BladderPatch | 7 | 39 | 4 | 39.067 |
| 21 | Implantation | 7 | 13 | 8 | 13.133 |
| 22 | Stenting | 8 | 9 | 3 | 9.05 |
| 23 | BladderPatch | 8 | 37 | 27 | 37.45 |
| 24 | Implantation | 8 | 13 | 5 | 13.083 |
| 25 | Stenting | 9 | 8 | 16 | 8.267 |
| 26 | BladderPatch | 9 | 39 | 38 | 39.633 |
| 27 | Implantation | 9 | 11 | 36 | 11.6 |
| 28 | Stenting | 10 | 8 | 34 | 8.567 |
| 29 | BladderPatch | 10 | 38 | 25 | 38.417 |
| 30 | Implantation | 10 | 11 | 14 | 11.233 |
| 31 | Stenting | 11 | 8 | 9 | 8.15 |
| 32 | BladderPatch | 11 | 33 | 23 | 33.383 |
| 33 | Implantation | 11 | 12 | 26 | 12.433 |
| 34 | Stenting | 12 | 7 | 28 | 7.467 |
| 35 | BladderPatch | 12 | 35 | 14 | 35.233 |
| 36 | Implantation | 12 | 12 | 17 | 12.283 |
| 37 | Stenting | 13 | 8 | 4 | 8.067 |
| 38 | BladderPatch | 13 | 33 | 56 | 33.933 |
| 39 | Implantation | 13 | 11 | 34 | 11.567 |
| 40 | Stenting | 14 | 7 | 17 | 7.283 |
| 41 | BladderPatch | 14 | 34 | 23 | 34.383 |
| 42 | Implantation | 14 | 10 | 57 | 10.95 |
| 43 | Stenting | 15 | 8 | 32 | 8.533 |
| 44 | BladderPatch | 15 | 34 | 31 | 34.517 |
| 45 | Implantation | 15 | 11 | 6 | 11.1 |
| 46 | Stenting | 16 | 7 | 44 | 7.733 |
| 47 | BladderPatch | 16 | 32 | 48 | 32.8 |
| 48 | Implantation | 16 | 9 | 43 | 9.717 |
| 49 | Stenting | 17 | 8 | 7 | 8.117 |
| 50 | BladderPatch | 17 | 35 | 8 | 35.133 |
| 51 | Implantation | 17 | 10 | 14 | 10.233 |
| 52 | Stenting | 18 | 7 | 56 | 7.933 |
| 53 | BladderPatch | 18 | 33 | 32 | 33.533 |
| 54 | Implantation | 18 | 9 | 58 | 9.967 |
| 55 | Stenting | 19 | 7 | 51 | 7.85 |
| 56 | BladderPatch | 19 | 34 | 57 | 34.95 |
| 57 | Implantation | 19 | 9 | 23 | 9.383 |
| 58 | Stenting | 20 | 8 | 18 | 8.3 |
| 59 | BladderPatch | 20 | 32 | 8 | 32.133 |
| 60 | Implantation | 20 | 10 | 26 | 10.433 |
| 61 | Stenting | 21 | 8 | 3 | 8.05 |
| 62 | BladderPatch | 21 | 32 | 55 | 32.917 |
| 63 | Implantation | 21 | 10 | 13 | 10.217 |
| 64 | Stenting | 22 | 8 | 27 | 8.45 |
| 65 | BladderPatch | 22 | 29 | 44 | 29.733 |
| 66 | Implantation | 22 | 9 | 56 | 9.933 |
| 67 | Stenting | 23 | 8 | 12 | 8.2 |
| 68 | BladderPatch | 23 | 32 | 13 | 32.217 |
| 69 | Implantation | 23 | 9 | 14 | 9.233 |
| 70 | Stenting | 24 | 8 | 6 | 8.1 |
| 71 | BladderPatch | 24 | 30 | 7 | 30.117 |
| 72 | Implantation | 24 | 10 | 5 | 10.083 |
| 73 | Stenting | 25 | 7 | 34 | 7.567 |
| 74 | BladderPatch | 25 | 29 | 28 | 29.467 |
| 75 | Implantation | 25 | 9 | 27 | 9.45 |
| 76 | Stenting | 26 | 8 | 23 | 8.383 |
| 77 | BladderPatch | 26 | 31 | 24 | 31.4 |
| 78 | Implantation | 26 | 10 | 18 | 10.3 |
| 79 | Stenting | 27 | 7 | 58 | 7.967 |
| 80 | BladderPatch | 27 | 30 | 46 | 30.767 |
| 81 | Implantation | 27 | 9 | 24 | 9.4 |
| 82 | Stenting | 28 | 8 | 21 | 8.35 |
| 83 | BladderPatch | 28 | 30 | 13 | 30.217 |
| 84 | Implantation | 28 | 9 | 48 | 9.8 |
| 85 | Stenting | 29 | 7 | 53 | 7.883 |
| 86 | BladderPatch | 29 | 32 | 16 | 32.267 |
| 87 | BladderPatch | 30 | 30 | 27 | 30.45 |
| 88 | BladderPatch | 31 | 29 | 56 | 29.933 |
